# Musculoskeletal Manifestations in Patients With Bardet-Biedl Syndrome: A Report of Two Cases

**DOI:** 10.7759/cureus.41963

**Published:** 2023-07-16

**Authors:** Gabriela M Arroyo Gonzalez, Natalio Izquierdo

**Affiliations:** 1 Medicine, School of Medicine, Medical Sciences Campus, University of Puerto Rico, San Juan, PRI; 2 Surgery, School of Medicine, Medical Sciences Campus, University of Puerto Rico, San Juan, PRI

**Keywords:** case report, osteoarthritis, coxa vara, musculoskeletal manifestations, retinitis pigmentosa, polydactyly, bardet-biedl syndrome

## Abstract

We report two patients with musculoskeletal manifestations as part of the Bardet-Biedl syndrome. The first patient (case 1) was born with polydactyly and later diagnosed with coxa vara. He had homozygous pathogenic mutation in the BBS1 gene of the variant c.1645G>T (p.Glu459*). The second patient (case 2) had nyctalopia and progressive vision worsening had osteoarthritis symptoms. He had a heterozygous mutation in the BBS1 gene of the variant c.1169T>G (p.Met390Arg). Although polydactyly is the most prevalent musculoskeletal association in patients with the syndrome, co-management of the musculoskeletal manifestations remains of utmost importance in patients with the syndrome.

## Introduction

Bardet-Biedl syndrome (BBS) is a primary ciliopathy with variable penetration, phenotypic variability, and multisystem affection, such as rod-cone dystrophy, obesity, renal abnormalities, cognitive impairment, and hypogonadism [1]. For this reason, patients with this syndrome show a wide variety of clinical manifestations, including retinitis pigmentosa, obesity, renal abnormalities, and hypogonadism [2,3]. Patients show signs and symptoms of the disease throughout the first two decades of life [4]. The syndrome is inherited as an autosomal recessive trait [5]. There are several genes associated with the Bardet-Biedl syndrome [5]. In Puerto Rico, the most common gene leading to the syndrome is BBS1 [6].

Previous studies have reported on a variety of musculoskeletal manifestations in patients with the syndrome [7]. These include polydactyly, scars, and remnants of amputated fingers, short and broad bones, flat joint surfaces, irregular ulnar length, skull deformities, hip dysplasia, and short stature [2,7]. Kaushik et al. have demonstrated the impact of the BBS mutation on chondrocytic primary cilium and how it affects cartilage maintenance, oftentimes leading to osteoarthritis [8]. Sheffield et al. reported an increased presence of pro-inflammatory markers and cartilage-destructive proteases in other mammals [9]. Here, we report two patients who had diverse musculoskeletal manifestations as part of the BBS.

## Case presentation

Case 1

**Figure 1:**
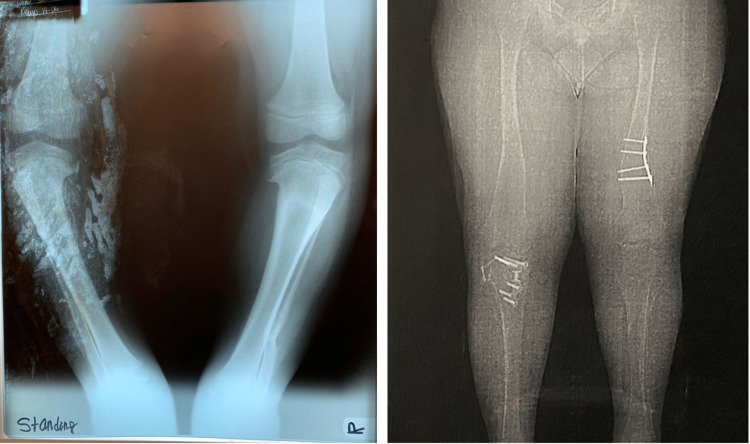
X-ray of case 1 before surgical intervention (left) versus CT scanogram after surgical intervention (right).

A 23-year-old male patient had a chief complaint of progressive worsening visual symptoms. He had poor visual acuity in both eyes (OU) since four years of age, night blindness OU starting at 19 years of age, and progressive loss of peripheral vision OU. The patient reported being born with an extra digit in each extremity, which was removed during the neonatal period. He was diagnosed with coxa vara and underwent surgery as shown in Figure 1. When asked about the educational level achieved, the mother stated that he required special education in the past. Upon physical examination, the patient had truncal obesity and a BMI of 52.8.

**Figure 2:**
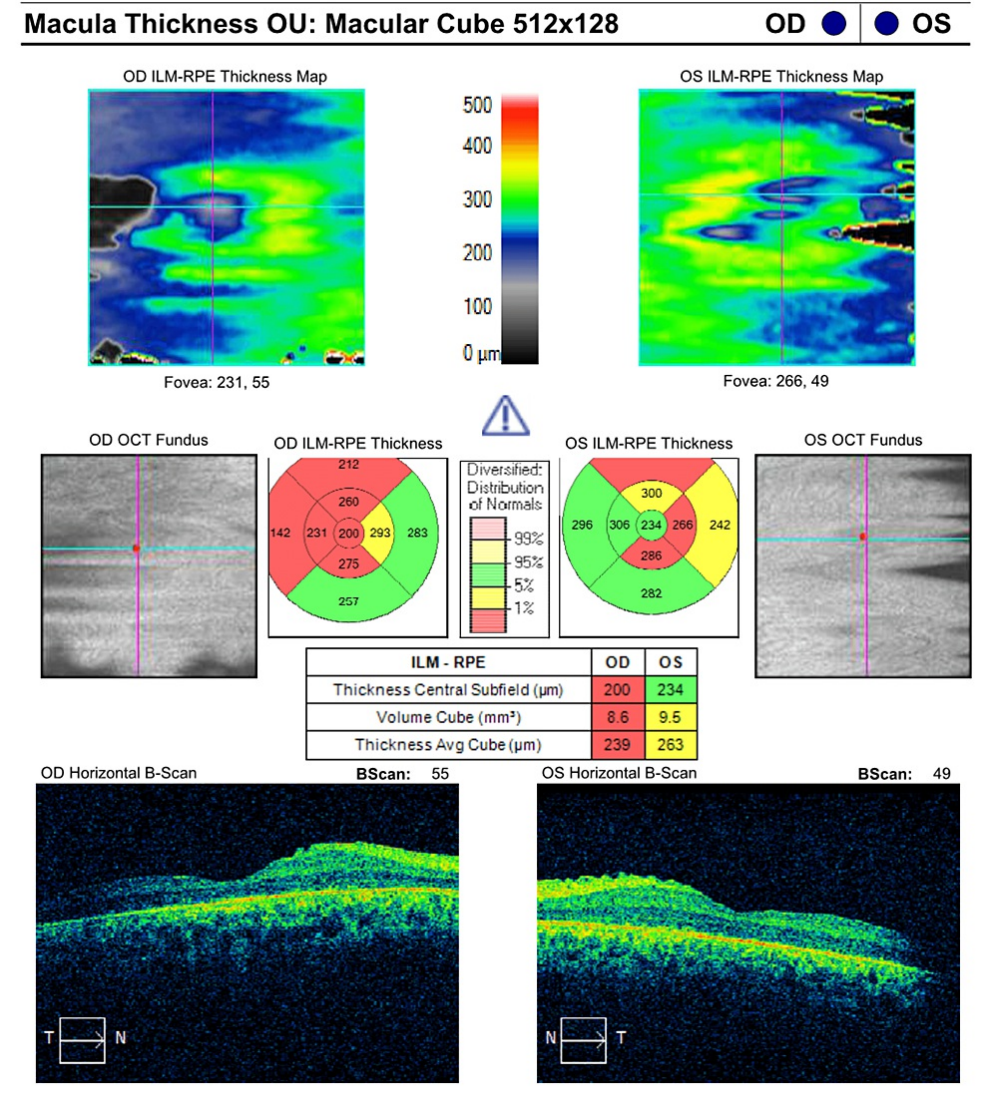
Ophthalmic evaluation of patient one. OD: right eye; OCT: optical coherence tomography; ILM: internal limiting membrane; RPE: retinal pigment epithelium; OU: both eyes; OS: left eye

The patient underwent a comprehensive ophthalmic evaluation as shown in Figure 2. Best corrected visual acuity was 20/800 in both eyes. Refraction was -4.50 +5.50 × 90° and -4.00 +5.50 × 90° in the right and left eye, respectively. Infrared fundus photography (Optos, Inc.) showed pale optic nerves, attenuated vessels, mid-peripheral bone spicules, and pigmentary macular changes, such as foveal reflex attenuation. Patient’s macular thickness is 196 and 232 µm in the right (OD) and left eye (OS), respectively. Total macular volume was 9.1 mm³ and 9.4 mm³ in the right and left eye, respectively. Visual field testing (30-2 Carl Zeiss Meditec, Inc.) showed a mean deviation of -31.65 dB (p<0.5%) in the OD and -31.89 dB (p<0.5%) in the OS. Photopic and scotopic electroretinogram (ERG) amplitudes were reduced more so in the OS than in OD, which is consistent with progressive rod-cone dystrophy.

A clinical diagnosis of retinitis pigmentosa (RP) as part of the syndrome was done. A genetic test was performed on a saliva sample, which revealed that the BBS1 gene had a homozygous pathogenic mutation in the variant c.1645G>T (p.Glu459*), as determined by gene sequencing and deletion/duplication analysis using next-generation sequencing (NGS) technology from Invitae Corporation in San Francisco, California. Patient was referred for genetic counseling.

Case 2

A 16-year-old male patient had a chief complaint of nyctalopia and worsening visual acuity. He also had complaints of joint pain consistent with osteoarthritis. The patient was born with no extra digits. Upon physical examination, the patient had truncal obesity and a BMI of 29.0 (overweight).

**Figure 3:**
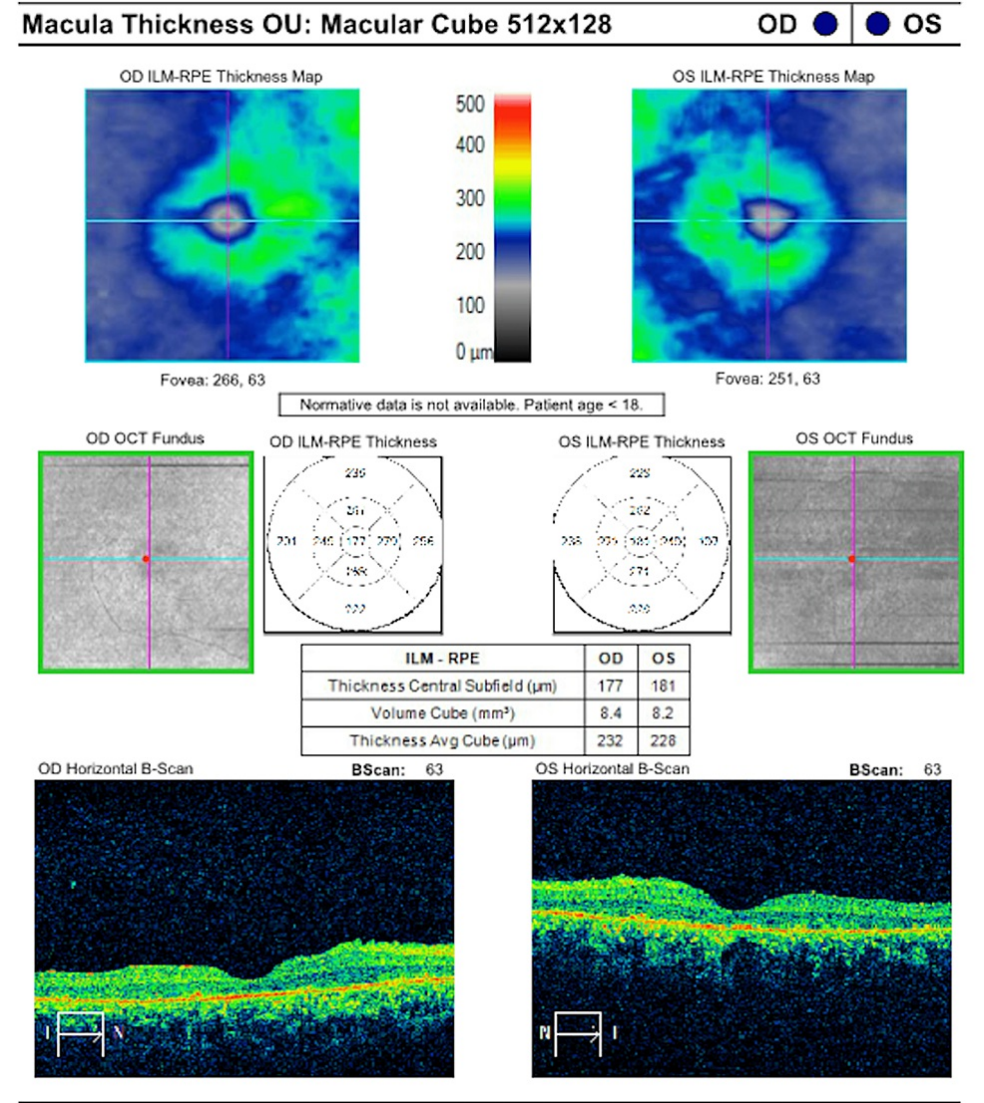
Ophthalmic evaluation of case 2. OD: right eye; OCT: optical coherence tomography; ILM: internal limiting membrane; RPE: retinal pigment epithelium; OU: both eyes; OS: left eye

The patient underwent a comprehensive ophthalmic evaluation as shown in Figure 3. Best corrected visual acuity was 20/50 and 20/60 in the right and left eye, respectively. Refraction was -1.00 +3.00 × 110° and -0.75 +2.75 × 85° in the right and left eye, respectively. The patient had a pale optic nerve, attenuated vessels, mid-peripheral bone spicules, and pigmentary macular changes, such as foveal reflex attenuation. Upon macular optical coherence tomography (Carl Zeiss Meditec, Inc.) the patient had macular thickness of 177 µm and 181 µm in the right and left eye, respectively. Total macular volume was 8.4 mm³ and 8.2 mm³ in the right and left eye, respectively. Visual field testing (30-2 Carl Zeiss Meditec, Inc.) showed a mean deviation of -26.85 dB (p<0.5%) and -27.45 dB (p<0.5%) in the right and left eye, respectively. Full-field ERG (LKC Technologies, Inc.) results showed abnormal scotopic and photopic response, consistent with progressive rod-cone dystrophy.

A clinical diagnosis of retinitis pigmentosa (RP) as part of the syndrome was reached and a saliva sample was sent for genetic testing. Using next-generation sequencing (NGS) technology from Invitae Corporation, San Francisco, CA, gene sequencing and deletion/duplication analysis were performed, revealing a heterozygous mutation in the variant c.1169T>G (p.Met390Arg) of the BBS1 gene. The patient was referred for genetic counseling.

## Discussion

Both case 1 and case 2 showcased the typical rod-cone dystrophy which characterizes the BBS [1]. Both also had central truncal obesity, while only the first patient (case 1) voiced the need for special education growing up; traits that add on to the diagnosis of the syndrome [1].

When the ophthalmologists Laurence and Moon first referenced the BBS in 1866, they described a variety of ciliopathies, including the syndrome [10]. What later went on to distinguish the BBS from other similar ophthalmic pathologies with rod-cone dystrophy, was the presence of polydactyly as well as other dystrophic extremities [10].

Previous studies have reported on the impact of diverse mutations of the BBS on the phenotypic manifestations of patients with the syndrome [11]. A zebrafish model with diverse knockdown BBS genes was used to demonstrate the effects on fins’ skeletal elements [11]. Further studies found a significant difference in cartilage thickness in BBS1 mice compared to wild type [9].

In our report, case 1 had polydactyly and coxa vara, which were both solved with surgery as shown in Figure 1. The second patient (case 2) was born without polydactyly but reported osteoarthritis. In our study, both patients had musculoskeletal manifestations. These findings are compatible with studies done by Motzkin et al. [2,8]. Previous studies by Kaushik et al. reported articular cartilage abnormalities consistent with early signs of osteoarthritis [8]. Case 2 had osteoarthritis. His findings are compatible with the study by Kaushik et al. [8].

Phenotypic variation in these patients may be due to their genetic differences. The first patient (case 1) has a homozygous pathogenic mutation in the BBS1 gene with variant c.1645G>T (p.Glu459*). On the other hand, the second patient (case 2) has a heterozygous mutation in the BBS1 gene with variant c.1169T>G (p.Met390Arg). The heterozygosity of case 2 and the particular gene variant could explain why his musculoskeletal manifestations are milder than those of case 1.

While musculoskeletal manifestations are not typically the focus of the BBS, they can help distinguish the syndrome from other rod-cone dystrophies in a clinical setting. These manifestations also contribute to the difficulties patients with the BBS face.

Limitations of the study include a small sample size due to the scarcity of patients with the syndrome. The prevalence of the syndrome is around 1/125,000 [12]. Further studies will elucidate the prevalence of musculoskeletal manifestations in patients with the BBS.

## Conclusions

A variety of musculoskeletal symptoms and signs may occur in patients with this syndrome. Both our patients had musculoskeletal signs and symptoms. Although polydactyly is the most prevalent musculoskeletal association in patients with the syndrome, further studies to evaluate other musculoskeletal findings in patients with the syndrome are warranted. Truncal obesity associated with polyphagia occurs in patients with the syndrome. An increased body mass index (BMI) may worsen musculoskeletal signs and symptoms in patients with the syndrome. Patients with the syndrome may benefit from multidisciplinary co-management. A multidisciplinary approach to multisystemic manifestations will improve patients’ quality of life.
